# Comparison of Calibration Methods for Tristimulus Colorimeters

**DOI:** 10.6028/jres.112.010

**Published:** 2007-06-01

**Authors:** James L. Gardner

**Affiliations:** National Measurement Institute, Lindfield, Australia 2070

**Keywords:** radiometry colorimetry, tristimulus colorimeter, uncertainty

## Abstract

Uncertainties in source color measurements with a tristimulus colorimeter are estimated for calibration factors determined, based on a known source spectral distribution or on accurate measurements of the spectral responsivities of the colorimeter channels. Application is to the National Institute of Standards and Technology (NIST) colorimeter and an International Commission on Illumination (CIE) Illuminant A calibration. Detector-based calibration factors generally have lower uncertainties than source-based calibration factors. Uncertainties are also estimated for calculations of spectral mismatch factors. Where both spectral responsivities of the colorimeter channels and the spectral power distributions of the calibration and test sources are known, uncertainties are lowest if the colorimeter calibration factors are recalculated for the test source; this process also avoids correlations between the CIE Source A calibration factors and the spectral mismatch factors.

## 1. Introduction

Tristimulus colorimeters are filter radiometers whose responses mimic the CIE 1931 color-matching distributions $\bar{x}$, $\bar{y}$, $\bar{z}$, as a function of wavelength [1]. The outputs of these radiometers are then proportional to the *X, Y, Z* tristimulus values so that values of various quantities used to describe color can be derived. Tristimulus colorimeters are usually calibrated by recording responses to a lamp whose spectral irradiance, or at least the relative spectral power distribution, is known. It was recently suggested [2] that lower uncertainties could be obtained by calibrating the responses of the colorimeter filter radiometers (channels) on a high-accuracy system such as the NIST laser-based Spectral Irradiance Comparison with Uniform Sources (SIRCUS facility) [3]. In this paper we compare uncertainties of tristimulus values and color quantities measured with colorimeters calibrated either on a detector- or the conventional source-based method. All the color quantities are related to the tristimulus values—here we deal only with the quantities of most interest in source colorimetry, (*x,y*) chromaticity values and correlated color temperature (CCT).

The $\bar{x}$ color-matching function has two peaks, separated by a region of almost-zero response near 504 nm. Modern colorimeters [4] separate the *X* channel into short- and long- wavelength parts and we assume this practice below, with the *X* tristimulus response being given as the sum of the *X*_1_ (short wavelength) and *X*_2_ (long wavelength) parts. Each filter radiometer produces an output current or voltage proportional to the tristimulus responses, a product of the source spectrum and the realized color matching functions. True tristimulus values are products of the source spectrum with the color-matching functions themselves. Calibration factors *k* are multipliers for the measured currents to convert to true tristimulus values. Ignoring scaling factors such as *k*_m_ (maximum luminous efficacy for conversion of radiometric quantities to photometric quantities) and gains, the calibration factors for the 4 channels are given by

$$\begin{array}{l} k_{x_{1}}=\frac{\sum E_{n}{\bar{x}}_{1,n}}{\sum E_{n}s_{x}{}_{{}_{1,n}}}\\ k_{x_{2}}=\frac{\sum E_{n}{\bar{x}}_{2,n}}{\sum E_{n}s_{x}{}_{{}_{2,n}}}\\ k_{y}=\frac{\sum E_{n}{\bar{y}}_{n}}{\sum E_{n}s_{y,n}}\\ k_{z}=\frac{\sum E_{n}{\bar{z}}_{n}}{\sum E_{n}s_{z,n}} \end{array}$$ (1)

where the *n* subscript denotes wavelength, *E* is the spectral irradiance and *s* is the spectral responsivity of a channel denoted by the leading subscript. The sum form shown assumes equal wavelength spacing between the points. In the examples below, an extra term has been used to account for variable spacing in the calculation of the integrated response of each channel. When calculating uncertainty components for the measured responsivity values, the source spectral distributions are calculated at the measurement wavelengths for the responsivity. When calculating uncertainty components for the source measurements, the responsivity values are interpolated to an evenly-spaced 5 nm grid. Such interpolation means that correlations introduced by interpolation can be ignored [5].

In many equations given below, only the expression for *X*_1_ is given; the remaining expressions are given by substituting the appropriate channel responsivity and color-matching function.

We want to estimate the uncertainties in source (*x,y*) chromaticity and CCT arising from uncertainties in the spectral responsivities and in the source spectral distributions, comparing these for detector- and source-based calibrations. There are two aspects to this. The first is that by tradition, colorimeters are calibrated for a CIE Source A spectral power distribution [6]. Hence we first estimate the accuracy of calibrations for this source for the two methods. The second stage is to estimate uncertainties for source distributions other than CIE Source A, for which in general both the responsivity distributions of the colorimeter channels and the spectral power distribution of the source must be known.

## 2. Uncertainty Propagation for (*x,y*) Chromaticity Values and CCT

Uncertainties are propagated as recommended by the International Organization for Standardization (ISO) [7]. CIE 1931 (*x,y*) chromaticities are given by

$$\begin{array}{l} x=\frac{X_{1}+X_{2}}{X_{1}+X_{2}+Y+Z}=\frac{X_{1}+X_{2}}{T_{xy}}\\ y=\frac{Y}{X_{1}+X_{2}+Y+Z}=\frac{Y}{T_{xy}} \end{array}$$ (2)

We estimate uncertainties (variances and covariances) for the tristimulus values, with *X* divided into its long and short wavelength parts (*X* = *X*_1_ + *X*_2_), then propagate uncertainties to those in chromaticity as

$$\begin{array}{l} \;\left[\begin{array}{cc} u^{2}(x) & u(x,y)\\ u(x,y) & u^{2}(y) \end{array}\right]=\\ Q_{\text{xy}}\left[\begin{array}{cccc} u^{2}(X_{1}) & u(X_{1},X_{2}) & u(X_{1},Y) & u(X_{1},Z)\\ u(X_{1},X_{2}) & u^{2}(X_{2}) & u(X_{2},Y) & u(X_{2},Z)\\ u(X_{1},Y) & u(X_{2},Y) & u^{2}(Y) & u(X,Y)\\ u(X_{1},Z) & u(X_{2},Z) & u(X,Y) & u^{2}(Z) \end{array}\right]Q_{\text{xy}}^{T} \end{array}$$ (3)

where **Q**xy is the matrix of sensitivity coefficients

$$\begin{array}{l} Q_{\text{xy}}=\left[\begin{array}{cccc} \frac{\partial x}{\partial X_{1}} & \frac{\partial x}{\partial X_{2}} & \frac{\partial x}{\partial Y} & \frac{\partial x}{\partial Z}\\ \frac{\partial y}{\partial X_{1}} & \frac{\partial y}{\partial X_{2}} & \frac{\partial y}{\partial Y} & \frac{\partial y}{\partial Z} \end{array}\right]\\ =\frac{1}{T_{\text{xy}}}\left[\begin{array}{cccc} 1-x & 1-x & -x & -x\\ -y & -y & 1-y & -y \end{array}\right] \end{array}$$ (4)

A similar process is used to estimate uncertainties in (*u,v*) chromaticity and the covariance between *u* and *v* [8]. Uncertainties in CCT are then propagated from those values [9,10].

We treat random and systematic uncertainties by component, using signed uncertainties for the systematic effects [8,11]; in practice, the sign is only important for systematic wavelength components. The total uncertainty in a given quantity can be found either by adding the variances and covariances for each independent effect in the tristimulus variance-covariance matrix in Eq. (3), or by separately propagating each component to that quantity; the latter method is preferable because it shows the relative importance of the various components. Systematic components that scale all spectral values equally are ignored, as they do not contribute to uncertainty in chromaticity. All values of uncertainty quoted in this paper are standard uncertainties, for a coverage factor *k* = 1.

### 2.1 Illuminant A Calibration Factors

#### 2.1.1 Detector-Based Calibration

We measure the irradiance responsivity of each of the channels as a function of wavelength. The source distribution can be taken as the exact distribution for CIE Illuminant A with no uncertainty. Only the denominators of Eqs. (1), that is, the calculated tristim-ulus responses, contribute to the uncertainty. Sensitivity coefficients for the dependence of the calibration factors on the responsivity values are of the form

$$\frac{\partial k_{x_{1}}}{\partial s_{x_{1},n}}=\frac{k_{x_{1}}}{\sum I_{A,n}s_{x_{1,n}}}I_{A,n}$$ (5)

where *I*A, *n* is the spectral irradiance of CIE Illuminant A at the *n*th wavelength.

For random components of the responsivity measurement, uncertainties of the calibration factors are given by

$$u^{2}(k_{x_{1}})={\left(\frac{k_{x_{1},n}}{\sum I_{A,n}s_{x_{1},n}}\right)}^{2}\sum I_{A,n}{}^{2}u^{2}\left(s_{x_{1},n}\right)$$ (6)

with similar expressions for the other channels. As these values are independent for random components, covariances between the tristimulus values are zero.

For systematic components of the responsivity measurement,

$$u_{s}(k_{x_{1}})=\frac{k_{x_{1}}}{\sum I_{A,n}s_{x_{1},n}}\sum I_{A,n}u_{s}(s_{x_{1},n})$$ (7)

For systematic components, the values of spectral responsivity are fully correlated, as are the tristimulus values, and hence covariances between the tristimulus values for systematic components are given by

$$\begin{array}{l} u(k_{x_{1}},k_{x_{2}})=u_{s}(k_{x_{1}})u_{s}(k_{x_{2}})\\ u(k_{x_{1}},k_{y})=u_{s}(k_{x_{1}})u_{s}(k_{y})\\ u(k_{x_{1}},k_{z})=u_{s}(k_{x_{1}})u_{s}(k_{z})\\ u(k_{x_{2}},k_{y})=u_{s}(k_{x_{2}})u_{s}(k_{y})\\ u(k_{x_{2}},k_{z})=u_{s}(k_{x_{2}})u_{s}(k_{z})\\ u(k_{y},k_{z})=u_{s}(k_{y})u_{s}(k_{z}) \end{array}$$ (8)

For uncertainty components of the calibration factors, relative uncertainties (variances and covariances) of the tristimulus values and the calibration factors are the same. Hence

$$u_{s}(X_{1})=\frac{X_{1}}{\sum I_{A,n}s_{x_{1},n}}\sum I_{A,n}u_{s}\left(s_{x_{1},n}\right)$$ (9)

and we can determine equivalent uncertainties of colorimetric quantities for a true CIE Illuminant A source measured with the colorimeter. Uncertainties in tristimulus values are found by replacing each calibration factor in Eqs. (6) and (7) with its related tristimulus value.

**Fig. 1:**
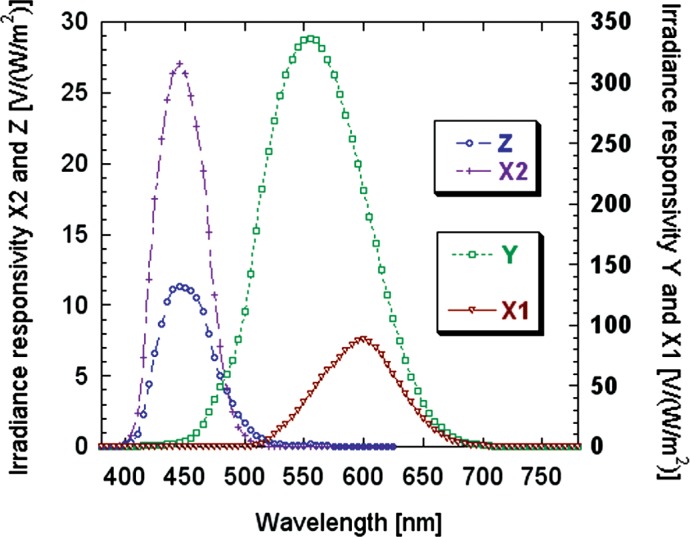
Spectral responsivity of the NIST colorimeter channels compared to the CIE color-matching functions (dashed lines). (Courtesy G. Eppeldauer, NIST)

The NIST tristimulus colorimeter with accurate matching to the CIE color matching functions (shown in Fig. 1) and the SIRCUS facility for spectral irradiance calibration are described elsewhere [3]. SIR-CUS is a laser-based facility and wavelength uncertainties are negligible, in contrast to calibrations using a conventional monochromator and broad-band source. Three uncertainty components from the calibration were used to propagate uncertainties of (*x,y*) chromaticity and CCT for CIE Illuminant A with the results shown in Table 1. The offset uncertainties shown in the table were estimated from the short-wavelength tail of the *X*_1_ responsivity distribution, where true signals were assumed to be zero.

Note that the accuracy of the calibration is independent of the matching of the responsivity functions to the CIE color-matching functions; this matching becomes important when the colorimeter is used to measure sources other than true Illuminant A. The levels shown in Table 1 would only be realized in a measurement of a CIE Source A lamp if the lamp was perfectly stable and no amplifier noise or drift was present. If we assume that each amplifier channel has a random noise level of 0.1 % of the signal current, equivalent uncertainties in (*x,y*) chromaticity and CCT would be (0.00040, 0.00053) and 1.7 K, respectively; these must be added in quadrature to the uncertainty values shown in the table. In practice, fluctuations in the lamp output due to lamp current variations correlate the signal currents. The correlation coefficients can be calculated for random lamp fluctuations [12,13]. Correlations will be increased if the lamp output is subject to drift. The correlations due to the combined lamp fluctuations and amplifier noise can also be determined from repeated measurements.

#### 2.1.2 Source-Based Calibration

A source-based calibration treats the source as the standard and the details of the responsivities of the channels are not required. The usual practice is to compare the lamp spectrum against a spectral irradiance standard and vary the lamp current until its measured spectrum has a correlated color temperature of 2856 K, that of CIE Illuminant A; calibration values are then given as the ratio of lamp tristimulus values to the measured currents. Note that correlations between these currents should also be measured, as discussed above.

We can propagate uncertainties from the spectral irradiance measurement to those of the tristimulus values, (*x,y*) chromaticity values and CCT [14] at the set value of CCT. (Uncertainties in CCT propagated from (*u,v*) chromaticities as described in Refs. [9,10] agree with those directly calculated as in Ref. [14]). Uncertainties arise from those of the primary standard and from components of the measurement process. The primary standard is usually a blackbody [15], but can be a lamp [16,17]; for each of these, spectral irradiance values are highly correlated [18], with the correlations diluted with each transfer to a working standard.

Table 2 shows typical uncertainty components for a spectral irradiance transfer from a blackbody-derived primary standard, along with the contribution to uncertainties in (*x,y*) chromaticity and CCT.

Relative uncertainties of the calibration factors (variances and covariances) are those estimated for the tristimulus values of the source.

These (*x,y*) uncertainties are larger than those for a colorimeter whose responsivities have been calibrated on SIRCUS. One difference between the two calculations is that the dominant random responsivity component does not lead to correlations between the tristimulus values; random components of the source measurement lead to correlations between the tristimulus values through the common dependence on the source spectral irradiance values. As for a detector-based calibration, uncertainties (variances and covariances) due to the signal current measurements should be added to the uncertainties of the calibration factors derived from the lamp.

## 3. Chromaticity Measurement of a General Source

The calibration factors measured for a particular source (usually CIE Source A) require multiplying by spectral mis-match factors to account for differences in the source spectrum from that of the calibration source, and for departures of the colorimeter spectral responsivities from those of the CIE color-matching functions. The general form of the mis-match multipliers is

$$F_{x_{1}}=\frac{\sum E_{A,n}s_{x_{1},n}}{\sum E_{A,n}{\bar{x}}_{{}_{1},n}}\frac{\sum E_{n}{\bar{x}}_{1,n}}{\sum E_{n}s_{x_{1},n}}$$ (10)

Estimation of the mis-match factors requires that both the channel spectral responsivities and the source spectral irradiance distributions be known. The source spectrum can vary widely, particularly for colored sources such as displays and LEDs. For comparison purposes, uncertainty calculations were made for the SIRCUS colorimeter measuring a 5500 K blackbody source. For 0.1 % random noise in the signal channels, the contributions to uncertainties in (*x,y*) chromaticity and CCT are (0.00017, 0.00018) and 1.2 K, respectively.

### 3.1 Responsivity Uncertainties

#### 3.1.1 Detector-Based Calibration

The mismatch factors are correlated to the original calibration factors through the responsivity terms common to both. Our interest is in estimating uncertainties in tristimulus values and hence chromaticity coordinates measured with the colorimeter; in effect we can multiply the original calibration factors by the mis-match factors to obtain new calibration factors and then propagate uncertainties through these new factors, avoiding the need to estimate the correlations:

$$k_{x_{1}}=\frac{\sum E_{n}{\bar{x}}_{1,n}}{\sum E_{n}s_{x_{1},n}}$$ (11)

where *E*_n_ is now the spectral irradiance of the source under test and not that of the calibration source. The propagation of responsivity uncertainties to those of the new calibration factors is as given in Sec. 2.1.1 with *I*_A,_*_n_* replaced by *E*_n_.

For the SIRCUS-calibrated colorimeter of Fig. 1 and a black-body source at 5500 K, the uncertainties in (*x,y*) chromaticity and CCT due to the calibration factors become (0.00005, 0.00005) and 0.8 K, respectively.

#### 3.1.2 Source-Based Calibration

The spectral mis-match factors are now independent of the calibration factors, or at most weakly correlated through a common base such as a cryogenic radiometer used to derive primary references. Sensitivity coefficients for the responsivity uncertainty components of the spectral mis-match factors are

$$\frac{\partial F_{x_{1}}}{\partial s_{x_{1},n}}=\frac{F_{x_{1}}}{\sum E_{A,n}s_{x_{1},n}}\left(E_{A,n}-E_{n}\frac{\sum E_{A,n}s_{x_{1},n}}{\sum E_{n}s_{x_{1},n}}\right)$$ (12)

The expression in the brackets is the difference between the test and calibration source spectral irradi-ances normalized to the same integral.

Relative uncertainties in the tristimulus values due to the responsivity uncertainties are those of the spectral-mis-match factors. For random components of the responsivity measurement,

$$\begin{array}{l} u^{2}(X_{1})=\\ {\left(\frac{X_{1}}{\sum E_{A,n}s_{x_{1},n}}\right)}^{2}\sum {\left(E_{A,n}-E_{n}\frac{\sum E_{A,n}s_{x_{1},n}}{\sum E_{n}s_{x_{1},n}}\right)}^{2}u^{2}(s_{x_{1},n})\\ =\sum {\left(E_{A,n}\frac{\sum E_{n}s_{x_{1},n}}{\sum E_{A,n}s_{x_{1},n}}-E_{n}\right)}^{2}u^{2}(s_{x_{1},n}) \end{array}$$ (13)

and the values of the different channels are uncorrelated.

For systematic components of the responsivity measurement,

$$u_{s}(X_{1})=\sum \left(E_{A,n}\frac{\sum E_{n}s_{x_{1,n}}}{\sum E_{A,n}s_{x_{1,n}}}-E_{n}\right)u_{s}(s_{x_{1,n}})$$ (14)

These values are fully correlated, with covariances given by the cross-products. Note that the sign of the normalized difference will change through the spectrum, and correlations between the tristimulus values may be positive or negative.

For the SIRCUS-calibrated tristimulus colorimeter, uncertainties in (*x,y*) chromaticity and CCT arising from the responsivity components of the spectral-mismatch factor are (0.00002, 0.00004) and 0.5 K, respectively. These terms are smaller than those for the detector-based calibration, but must be added in quadrature with the uncertainties from the original calibration.

From Eqs. (13) and (9) we can calculate the ratio of the tristimulus sensitivity coefficients for responsivity uncertainties for the lamp calibration method to those for the detector calibration method; for the *X*_1_ channel this is:

$$1-\frac{E_{n}}{\sum E_{n}s_{x_{1},n}}\frac{\sum E_{A,n}s_{x_{1,n}}}{E_{A,n}}$$ (15)

For CIE Source A, the ratios are zero. Each spectral value is normalised to its sum weighted by the color-matching functions, and it is the ratios of these normalised values that determines the ratio of sensitivities. For a blackbody distribution at 3300 K, the magnitude of the ratio averaged over the wavelength range 360 nm to 830 nm at 5 nm intervals is 0.29. This ratio becomes 1 at a temperature near 4950 K; at 6500 K, the average ratio is 1.54. The conclusion is that responsivity uncertainty contributions to the spectral mismatch are smaller for the source-based calibration method near the calibration point, but comparable as relative differences between the test spectrum and calibration spectrum increase. In practice, measurement of the colorimeter response spectra are likely to be made on a conventional monochromator based system at relatively low light levels, and uncertainties in responsivity will be much larger than those achieved in the SIRCUS calibration.

### 3.2 Source Spectral Irradiance Uncertainties

#### 3.2.1 Detector-Based Calibration

Sensitivity coefficients for the dependence of the new calibration factors on the source irradiance values are of the form

$$\frac{\partial k_{x_{1}}}{\partial E_{n}}=\frac{1}{\sum E_{n}s_{x_{1,n}}}\left({\bar{x}}_{1,n}-\frac{\sum E_{n}{\bar{x}}_{1,n}}{\sum E_{n}s_{x_{1},n}}s_{x_{1},n}\right)$$ (16)

It is the quality of the spectral match that determines the sensitivity to source variations—if the spectral match is exact, these terms are zero. Equivalent sensitivity coefficients for the tristimulus values are found by multiplying each term by the ratio of the tristimulus value to the calibration factor. For random components of the spectral irradiance measurement, we then have:

$$\begin{array}{l} u^{2}(X_{1})=\\ {\left(\frac{X_{1}}{\sum E_{n}{\bar{x}}_{1,n}}\right)}^{2}\sum {\left({\bar{x}}_{1,n}-s_{x_{1},n}\frac{\sum E_{n}{\bar{x}}_{1,n}}{\sum E_{n}s_{x_{1},n}}\right)}^{2}u^{2}(E_{n})\\ =\sum {\left({\bar{x}}_{1,n}\frac{\sum E_{n}s_{x_{1},n}}{\sum E_{n}{\bar{x}}_{1,n}}-s_{x_{1},n}\right)}^{2}u^{2}(E_{n}) \end{array}$$ (17)

Unlike the spectral responsivity random components, the tristimulus values are now correlated even for random components through the common source spectral dependence; these correlations are given by the cross-product of sensitivity coefficients and the variance at a given wavelength:

$$\begin{array}{l} u(X_{1},X_{2})=\\ \sum \left({\bar{x}}_{1,n}\frac{\sum E_{n}s_{x}{}_{{}_{1},n}}{\sum E_{n}{\bar{x}}_{1,n}}-s_{x_{1},n}\right)\left({\bar{x}}_{2,n}\frac{\sum E_{n}s_{x}{}_{{}_{2},n}}{\sum E_{n}{\bar{x}}_{2,n}}-s_{x_{2},n}\right)u^{2}(E_{n}) \end{array}$$ (18)

with similar expressions for the other terms.

For systematic components of the spectral irradiance measurements, individual spectral values are fully correlated, as are the tristimulus values.

$$u_{s}(X_{1})=\sum \left({\bar{x}}_{1,n}\frac{\sum E_{n}s_{x}{}_{{}_{1},n}}{\sum E_{n}{\bar{x}}_{1,n}}-s_{x_{1},n}\right)u_{s}(E_{n})$$ (19)

Correlations are as in Eq. (8).

For the SIRCUS-calibrated colorimeter of Fig. 1 and spectral irradiance uncertainties as in Table 2, equivalent uncertainties in (*x,y*) chromaticity and CCT are (0.00020, 0.00026) and 3.2 K, respectively.

#### 3.2.2 Source-Based Calibration

Sensitivity coefficients for the dependence of the mismatch factors on the source spectral irradiance are:

$$\frac{\partial F_{x_{1}}}{\partial E_{n}}=\frac{F_{x_{1}}}{\sum E_{n}{\bar{x}}_{1}{}_{,,n}}\left({\bar{x}}_{1,n}-s_{x_{1},n}\frac{\sum E_{n}{\bar{x}}_{1,n}}{\sum E_{n}s_{x}{}_{{}_{1},n}}\right)$$ (20)

Equivalent sensitivity coefficients for the tristimulus values are found by multiplying each term by the ratio of the tristimulus value to the calibration factor. Uncertainties in the tristimulus values arising from uncertainties in the source spectral irradiance are then identical to those of the previous section for a detector-based calibration.

In general, the source spectrum used at the time of calibration is not known and in calculating the spectral mismatch factor, the tabulated distribution for CIE Illuminant A is used rather than a CIE Source A distribution. The uncertainties of the spectral mis-match factor due to source uncertainties are then uncorrelated to the original source-based calibration and the total uncertainty is calculated by adding the variances and covariances of the calibration factors with those of the spectral mis-match factors.

Consider now the case where spectral mis-match factors are calculated knowing the CIE Source A spectral distribution used for determining the calibration factors, and its uncertainties. The calibration factor and mis-match correction for a given channel are then correlated. The simplest way of treating these correlations is to consider the uncertainties in the new calibration factor formed as the product of the two terms:

$$k_{x_{1}}{}^{\prime }=\frac{\sum E_{A,n}{\bar{x}}_{1,n}}{\sum E_{A,n}s_{x_{1,n}}}\frac{\sum E_{A,n}s_{x_{1,n}}}{\sum E_{A,n}{\bar{x}}_{1,n}}\frac{\sum E_{n}{\bar{x}}_{1,n}}{\sum E_{n}s_{x_{1,n}}}=\frac{\sum E_{n}{\bar{x}}_{1,n}}{\sum E_{n}s_{x_{1,n}}}$$ (21)

The new calibration factor is now equivalent to that of a detector-based calibration; uncertainties due to source components are identical to the above two cases, except that the uncertainty due to the source componentis now used alone and that of the original calibration is not added.

### 3.3 Comparison of a Tristimulus Colorimeter Measurement With Direct Calculation

In theory, color measurements for any source can be made by directly measuring the spectral irradiance distribution of the source and calculating the tristimulus values using the color-matching functions. In practice, accurate measurement of spectral irradiance distributions requires good quality reference standards and high-quality spectral equipment not suited to measurements in the field and not suited to low-brightness sources.

Direct calculation of color values is the basis of a source-based calibration. Uncertainties in (*x,y*) chromaticity and CCT for a 2856 K black-body source are given in Table 1; for a blackbody source at 5500 K, these uncertainties become (0.00033, 0.00047) and 14.6 K respectively, for the same spectral irradiance component uncertainties.

Source uncertainties dominate the CCT uncertainty of the recalculated calibration factor (Secs. 3.1.1 and 3.2.1). If we compare these uncertainties with those of a recalculated calibration factor knowing both the spectral responsivities and the spectral irradiance, the same accuracy in CCT can be achieved with the combined uncertainty components of spectral irradiance about 4 times higher than those listed in Table 2. Hence the spectral irradiance distribution may be able to be measured with a simpler system, such as an array spectrometer, to calculate the colorimeter responses. Note that here we have not dealt with systematic components such as scattered light and bandwidth that may affect the accuracy of a spectral measurement [19]. Similarly, the uncertainty of the measurement of spectral responsivity can be relaxed from that of the SIRCUS measurement without greatly degrading the accuracy of the calibration factors.

### 3.4 Alternative Tristimulus Colorimeter Calibration Schemes

Uncertainties can be minimized by calibrating a tris-timulus colorimeter with a source distribution that matches that of the application. One such scheme uses a spectrally-tuneable source to mimic the spectral distribution of the application [20]. For an exact match to the source spectral distribution, the uncertainty of the calibration is calculated as in Sec. 2.1.2. If the source match is not exact, spectral mis-match factors must be applied and their uncertainties estimated. If the match of the spectrometer responsivity functions to the color-matching functions is good, uncertainties in the mismatch factor are small and accurate measurements can be obtained. As in Sec. 3.2.2, the spectral distribution at the time of calibration is known and low uncertainties can be obtained by recalculating the calibration factors as in Eq. (21).

Another scheme applied to displays is to calibrate the colorimeter for a range of spectral distributions and form a matrix of calibration factors [21,22]; uncertainties for each of these can be estimated as above, but an individual application still requires an estimate of the mis-match factor to properly determine the accuracy of a particular measurement. Calculation of the mis-match factor requires the spectral responsivities of the channels. It is not always possible to access the current signals in a colorimeter or to have access to spectral responsivity equipment and standards. The matrix method or a spectrally tuneable source must then be applied, with uncertainties expanded to cover measurements on sources other than used for the calibration.

### 3.5 Colorimeter Quality Factors

The bracketed terms in Eqs. (12) and (16) determine the relative sensitivity of the tristimulus values to departures in the spectral dependence of the source compared to that used at the time of calibration and to departures in responsivity of the channels compared to the color-matching functions. For the latter, we can determine quality factors analogous to the photometric quality factor *f*_1_´:

$$f_{1,x^{\prime }}=\sum \left|{\bar{x}}_{n}-s_{x_{1},n}\frac{\sum E_{A,n}{\bar{x}}_{n}}{\sum E_{A,n}s_{x_{1},n}}\right|/\sum {\bar{x}}_{n}$$ (22)

where the two parts of the *X* channel have been summed, with similar expressions for the *Y* and *Z* channels.

These factors are indicators only - the smaller the number, the more tolerant the colorimeter is to variations in source spectrum compared to that at the time of calibration.

For the colorimeter shown in Fig. 1, the *f*_1_′ values are 2.7 %, 1.7 % and 3.6 % for the *X*, *Y* and *Z* channels, respectively. (In calculating these values, the channel responsivity values were interpolated to a 5 nm grid over the wavelength range 380 nm to 780 nm.) NIST has developed an iterative technique for refining color measurements on a tungsten source [2], where the CCT of the source is determined with the original calibration factors, then recalculated with a spectral mis-match factor based on that CCT value compared with the Illuminant A calibration. This technique gives good results, and is more accurate the better the values of the *f*_1_′ colorimeter [Y. Ohno, personal communication]. The technique is equally applicable to colorimeters whose calibration factors have been determined on either a source or detector basis, provided the respon-sivities of the channels are known.

## 4. Conclusion

Various calibration techniques for tristimulus colorimetry have been discussed. Table 3 summarizes the calculations of uncertainty components for the NIST colorimeter with calibration factors determined on a detector basis and a source basis, for the base calibration at a CIE Source A spectrum and for a black-body at 5500 K. This colorimeter reproduces the CIE color-matching distributions with high accuracy and the uncertainties for a colorimeter of lesser quality would be higher.

A clear conclusion is that if the spectral responsivity of the colorimeter channels is known and the source distribution is known, recalculating the calibration factors for a particular source leads to lower uncertainties in color quantities than applying a spectral-mis-match factor based on the CIE Illuminant A distribution.

If the spectral responsivities of the tristimulus channels are not known, the colorimeter should be calibrated on a source whose spectral power distribution matches that of the application.

## Figures and Tables

**Table 1:** Uncertainties in *(x,y*) chromaticities and CCT for a true CIE Illuminant A source measured with the NIST colori-meter propagated from uncertainty components for responsivity measurements on the SIRCUS facility

Responsivity Uncertainty Component	Component Value	*u*(*x*)	*u*(*y*)	*u*(CCT) (K)
Relative, random	0.0006	0.00010	0.00013	0.8
Offset, random (relative to *Y* maximum)	6 · 10^−6^	0.00001	0.00001	–
Offset, systematic (relative to *Y* maximum)	2 · 10^−5^	0.00001	0.00001	–
Total:		0.00011	0.00013	0.8

**Table 2:** Uncertainties in (*x,y*) chromaticities and CCT for a realized CIE Source A for typical spectral irradiance uncertainty components

Uncertainty Component	Type	Component Value	*u*(*x*)	*u*(*y*)	*u*(CCT) (K)
Reference 2800 K blackbody		2 K in temperature 0.9 correlation coefficient	0.00020	0.00014	3.12
Reference signal offset (noise)	Random	0.1 % of signal maximum	0.00013	0.00015	1.74
Reference signal offset (drift)	Systematic	0.03 % of signal maximum	0.00012	0.00008	1.25
Test signal offset (noise)	Random	0.1 % of signal maximum	0.00010	0.00012	1.43
Test signal offset (drift)	Systematic	0.03 % of signal maximum	0.00009	0.00006	0.98
Source noise (relative)	Random	0.1 % (in signal ratio)	0.00003	0.00003	0.60
Wavelength	Random	0.3 nm	0.00001	0.00000	0.09
Wavelength offset	Systematic	0.1 nm	0.00000	0.00000	0.01
Sum of all components			0.00030	0.00026	4.2

**Table 3:** Summary of uncertainty calculations: I = current component (random, 0.1 %); R = responsivity components (see Table 1); S = spectral irradiance components (see Table 2)

Illuminant A							
Detector calibration				Source calibration			
	I	R		S			

*u*(*x*)	0.00040	0.00011		0.00030			
*u*(*y*)	0.00053	0.00013		0.00026			
*u*(CCT)							
(K)	1.66	0.83		4.20			

5500 K blackbody source							

Detector calibration					Source calibration (mismatch Source A uncorrelated)		Source

					Mismatch		

	I	R	S	Base S	R	S	S

*u*(*x*)	0.00017	0.00005	0.00020	0.00035	0.00002	0.00020	0.00035
*u*(*y*)	0.00018	0.00005	0.00026	0.00047	0.00004	0.00026	0.00047
*u*(CCT)							
(K)	1.22	0.76	3.16	4.20	0.48	3.16	14.6

## References

[1] Colorimetry (2004). International Commission on Illumination.

[2] Eppeldauer G, Ohno Y (2006). Development of a NIST detector-based color temperature scale.

[3] Brown SW, Eppeldauer GP, Lykke KR (2006). Facility for Spectral Irradiance and Radiance Responsivity Calibrations using Uniform Sources (SIRCUS). Appl Opt.

[4] Schanda J, Eppeldauer G, Sauter G, Schanda J (2007). Tristimulus colour measurement of self-luminous sources. CIE Colorimetry: Understanding the CIE system.

[5] Gardner JL (2003). Uncertainties in interpolated spectral data. J Res Natl Inst Stand Technol.

[6] CIE Standard Illuminants for Colorimetry (1999). International Commission on Illumination.

[7] (1993). Guide to the Expression of Uncertainty in Measurement.

[8] Gardner JL, Schanda J (2007). Uncertainties in spectral colour measurement. CIE Colorimetry: Understanding the CIE system.

[9] Gardner JL (2000). Correlated color temperature—uncertainty and estimation. Metrologia.

[10] Fontecha J, Campos J, Corrons A, Pons A (2002). An analytical method for estimating the correlated color temperature uncertainty. Metrologia.

[11] Gardner JL, Parr A, Datla R, Gardner J (2005). Uncertainty estimates in radiometry. Optical Radiometry.

[12] Robertson AR (1967). Colorimetric significance of spectrophoto-metric errors. J Opt Soc Am.

[13] Gardner JL (2000). Uncertainty estimation in colour measurement. Color Res Application.

[14] Gardner JL (2006). Uncertainties in source distribution temperature and correlated colour temperature. Metrologia.

[15] Walker JH, Saunders RD, Jackson JK, McSparron DA (1987). Spectral irradiance calibrations. Natl Inst Stand Technol Spec Publ.

[16] Boivin LP, Gaertner AA (1992). Realization of a spectral irradiance scale in the near infrared at the National Research Council of Canada. Appl Opt.

[17] Karha P, Toivanen P, Manoochehri F, Ikonen E (1997). Development of a detector-based spectral irradiance scale in the 380–900 nm spectral range. Appl Opt.

[18] Gardner JL (2003). Correlations in primary spectral standards. Metrologia.

[19] Ohno Y, Schanda J (2007). Spectral colour measurement. CIE Colorimetry: Understanding the CIE system.

[20] Fryc I, Brown SW, Eppeldauer GP, Ohno Y (2005). LED-based spectrally tunable source for radiometric, photometric and colorimetric applications. Optical Engineering.

[21] Ohno Y, Hardis J (1997). Four-Color Matrix Method for Correction of Tristimulus Colorimeters.

[22] Ohno Y, Brown SW (1998). Four-Color Matrix Method for Correction of Tristimulus Colorimeters—Part 2.

